# Deciphering the Antibacterial Mode of Action of Alpha-Mangostin on *Staphylococcus epidermidis* RP62A Through an Integrated Transcriptomic and Proteomic Approach

**DOI:** 10.3389/fmicb.2019.00150

**Published:** 2019-02-06

**Authors:** Murugesan Sivaranjani, Katarzyna Leskinen, Chairmandurai Aravindraja, Päivi Saavalainen, Shunmugiah Karutha Pandian, Mikael Skurnik, Arumugam Veera Ravi

**Affiliations:** ^1^Department of Biotechnology, Alagappa University, Karaikudi, India; ^2^Department of Bacteriology and Immunology, Medicum, Research Programs Unit, Immunobiology Research Program, University of Helsinki, Helsinki, Finland; ^3^Division of Clinical Microbiology, Helsinki University Hospital, HUSLAB, Helsinki, Finland

**Keywords:** RNA-sequencing, LC-MS/MS, bactericidal, alpha-mangostin, cytoplasmic membrane

## Abstract

**Background:** Alpha-mangostin (α-MG) is a natural xanthone reported to exhibit rapid bactericidal activity against Gram-positive bacteria, and may therefore have potential clinical application in healthcare sectors. This study sought to identify the impact of α-MG on *Staphylococcus epidermidis* RP62A through integrated advanced omic technologies.

**Methods:** *S. epidermidis* was challenged with sub-MIC (0.875 μg/ml) of α-MG at various time points and the differential expression pattern of genes/proteins were analyzed in the absence and presence of α-MG using RNA sequencing and LC-MS/MS experiments. Bioinformatic tools were used to categorize the biological processes, molecular functions and KEGG pathways of differentially expressed genes/proteins. qRT-PCR was employed to validate the results obtained from these analyses.

**Results:** Transcriptomic and proteomic profiling of α-MG treated cells indicated that genes/proteins affected by α-MG treatment were associated with diverse cellular functions. The greatest reduction in expression was observed in transcription of genes conferring cytoplasmic membrane integrity (*yidC2, secA* and *mscL*), cell division (*ftsY* and *divlB*), teichoic acid biosynthesis (*tagG* and *dltA*), fatty-acid biosynthesis (*accB, accC, fabD, fabH, fabI*, and *fabZ*), biofilm formation (*icaA*) and DNA replication and repair machinery (*polA, polC, dnaE*, and *uvrA*). Those with increased expression were involved in oxidative (*katA* and *sodA*) and cellular stress response (*clpB, clpC, groEL*, and *asp23*). The qRT-PCR analysis substantiated the results obtained from transcriptomic and proteomic profiling studies.

**Conclusion:** Combining transcriptomic and proteomic methods provided comprehensive information about the antibacterial mode of action of α-MG. The obtained results suggest that α-MG targets *S. epidermidis* through multifarious mechanisms, and especially prompts that loss of cytoplasmic membrane integrity leads to rapid onset of bactericidal activity.

## Introduction

*Staphylococcus epidermidis* is one of the most important causes of nosocomial and community acquired infections (Gomes et al., 2014). It is often observed in the device related and surgical site infections, where the biofilm formation on implants and tissues further increases the treatment failure (Center for Disease Control Prevention [CDC], 2013; Danzmann et al., 2013; Gomes et al., 2014). In addition, infections associated with biofilms are persisting until the subsequent replacement or removal of implants, which causes distress to patients and lead to superfluous expenditure (Donlan and Costerton, 2002). The available antibiotic therapy can only kill planktonic cells, leaving the bacterial cells to grow within the biofilms continuously even after the termination of antibiotic therapy (Roper et al., 2000; Parra-Ruiz et al., 2012; Reiter et al., 2014). Alarmingly, the ability of biofilm to resist clearance by antibiotics increased the importance of a continuous search for novel antibacterial agents that target both planktonic and biofilm populations. Hence, new antibacterial agents are needed to combat biofilm mediated infections caused by *S. epidermidis*.

Throughout human history, plants have been the inexhaustible source of novel bioactive compounds with multitude of blockbuster therapeutic drugs, which have been derived directly or indirectly from various plants (Kinghorn et al., 2011; Newman and Cragg, 2012; Veeresham, 2012; Pohlit et al., 2013; Laallam et al., 2015; Satthakarn et al., 2015; Khan et al., 2018). Among the reported plant-derived bioactive compounds, alpha-mangostin (α-MG), a natural xanthone derived from the pericarp of *Garcinia mangostana* has been reported for various pharmacological properties that includes antibacterial, antifungal, anti-inflammatory, anticancer, and antioxidant activity (Ibrahim et al., 2016). α-MG elicits *in vitro* rapid bactericidal activity against several Gram-positive pathogens (Nguyen and Marquis, 2011; Koh et al., 2013; Sivaranjani et al., 2017). As reported by Koh et al. (2013) α-MG rapidly disintegrates the cytoplasmic membrane integrity of methicillin resistant *Staphylococcus aureus* (MRSA), which results in loss of cytoplasmic components. The multi-step resistance selection assay from previous studies suggested that Gram-positive pathogens do not develop resistance against α-MG (Koh et al., 2013; Sivaranjani et al., 2017). Most importantly, data from our previous study confirmed that α-MG effectively inhibits the onset of biofilm formation as well as disrupts the immature and mature biofilms of *S. epidermidis* RP62A biofilms, though the highest concentration of vancomycin was inefficient in killing the sessile cells of *S. epidermidis* RP62A (Sivaranjani et al., 2017). Similarly, Nguyen et al. (2014) reported that topical application of α-MG can effectively disrupt the development and structural integrity of *Streptococcus mutans* biofilm, which facilitates the mechanical clearance of cariogenic biofilms. Besides, several studies have demonstrated efficient methods to synthesize α-MG derivatives that also reflects the importance of α-MG and its derivatives in biological research (Matsumoto et al., 2004; Ha et al., 2009; Xu et al., 2013; Zou et al., 2013; Fei et al., 2014; Koh et al., 2015; Li et al., 2015; Koh et al., 2016). The potential bottleneck to develop α-MG as an effective antibacterial agent is the very limited understanding of the molecular mechanism of action of α-MG. Indeed, several studies have used omics techniques to elucidate the antibacterial mode of action of plant-derived compounds (Reddy et al., 2015; Dos Santos et al., 2016). Though, the rapid antibacterial mode of action of α-MG has been already investigated through *in vitro* and *in silico* approaches (Koh et al., 2013), integrated advanced omics technologies will further augment the current knowledge on the mode of action of α-MG. In the present study, we investigated the molecular mechanism of antibacterial activity of α-MG through an integrated transcriptomic and proteomic approach.

## Materials and Methods

### Bacterial Strain and Chemical

*Staphylococcus epidermidis* RP62A (ATCC 35984) was routinely grown in Luria-Bertani (LB; HiMedia, India) and was maintained in LB with 30% glycerol at -80°C. α-MG was purchased from Sigma-Aldrich (Catalog No.: M3824) and stock solution of 1 mg/mL was prepared in methanol.

### Antibacterial Assays

The minimum inhibitory concentration (MIC), minimum bactericidal concentration (MBC) and time kill kinetics assays were previously determined (Sivaranjani et al., 2017). The MIC and MBC values of α-MG were determined again to precede subsequent assays (Clinical Laboratory Standards Institute [CLSI], 2006). The spot assay was carried out to determine the antibacterial activity of α-MG on mid-log phase cultures. Different concentrations of α-MG [1.25 μg/mL (MIC), 0.875 μg/mL (0.7 MIC), 0.625 μg/mL (0.5 MIC), 0.3125 μg/mL (0.25 MIC)] were added to mid-log phase (∼2.5 × 10^8^ CFU/ml) cultures that were subsequently incubated at 37°C for 10 min. After incubation, 10 μl of serially diluted control (0.1% methanol) and α-MG treated samples were spotted on LB plates. The plates were incubated at 37°C for 18 h.

### RNA-Sequencing and Data Analysis

Overnight cultures of *S. epidermidis* were diluted in 1:100 in LB and were grown to mid-log phase (∼2.5 × 10^8^ CFU/ml) at 37°C. Bacterial cells were treated with 0.7 MIC (0.875 μg/ml) of α-MG or 0.1% methanol (solvent control) for 10 min. After incubation, 2 volumes of RNAprotect Bacteria Reagent (Qiagen, Hilden, Germany) were added to one volume of bacterial cultures. Then, the cells were pelleted by centrifugation, resuspended in 200 μl of TE buffer containing 15 mg/ml of lysozyme and 10 μl of proteinase K and incubated at 37°C for 30 min. After incubation, zirconia beads (50 mg; 0.1 mm) were added to lyse the cells using FastPrep 24 instrument (Qbiogene, Heidelberg, Germany) and the total RNA was extracted using RNeasy Mini kit (Qiagen, Hilden, Germany). To remove genomic DNA, on-column DNA digestion was done using RNase-Free DNase set (Qiagen, Hilden, Germany). The RNA integrity was analyzed using LabChip GXII Touch HT (PerkinElmer, United States). The ribosomal RNA (rRNA) was depleted using Ribo-Zero rRNA Removal kit for Gram-positive bacteria (Illumina, San Diego, CA, United States). Sequencing library was prepared as described before with modifications (Macosko et al., 2015). The libraries were sequenced in Biomedicum Functional Genomics Unit (Helsinki, Finland). Reverse transcription of mRNA was performed using specially designed primers. Similarly to the method described in Macosko et al. (2015), all the primers shared a common sequence that served as a PCR handle. Additionally, the primers contained known 12 bp barcode (unique for each sample) and different random unique molecular identifiers (UMIs) (Macosko et al., 2015). The poly (T) regions of the oligos were replaced by random hexamers to enable the capturing of the bacterial mRNA particles. The reverse transcription, PCR amplification of the cDNA and tagmentation were performed as described by Macosko et al. (2015). Paired-end sequencing was performed on NextSeq500 sequencer (Illumina) using the sequencing primers (Macosko et al., 2015). Sequencing in 75 cycles produced 20 bp Read 1 (bases 1–12 sample barcode, bases 13–20 UMI) and 55 bp Read 2 (sequence derived from the bacterial mRNA). The raw sequencing reads were analyzed using the Dropseq Core Computational Protocol (Macosko et al., 2015). The obtained sequencing reads were aligned against the strain *S. epidermidis* RP62A (ATCC 35984) genome using the STAR aligner (Dobin et al., 2013). The edgeR, a Bioconductor package was used to obtain the list of differentially expressed genes (Robinson et al., 2010). The RNA sequence data has been deposited to NCBI Gene Expression Omnibus (Accession No.: GSE113302).

### Quantitative Proteomics

The mid-exponential growth phase (∼2.5 × 10^8^ CFU/ml) cultures were treated with 0.7 MIC (0.875 μg/ml) of α-MG or 0.1% methanol (solvent control) for 10 and 30 min. After incubation, α-MG treated and untreated cells were harvested by centrifugation at 3500 × *g*, washed with sterile PBS and resuspended in 1 ml of lysis buffer (0.1% RapiGest, 8 M urea, 100 mM ammonium bicarbonate). The cell suspension was sonicated for 10 min (Branson Sonifier 450; 30 s running, 30 s pause, pulsed mode 30%) and stored at -70°C. Prior to trypsin digestion of proteins, the protein samples were reduced with Tris (2-carboxyethyl) phosphine and alkylated with iodoacetamide. Subsequently, tryptic peptide digests were purified using C18 reversed-phase columns (Varjosalo et al., 2013) and the mass spectrometric analysis was performed in Orbitrap Elite ETD mass spectrometer (Thermo Scientific), using Xcalibur version 2.7.1 coupled with a Thermo Scientific nLCII nanoflow HPLC system. MS peak extraction and subsequent protein identification was analyzed using Proteome Discoverer software (Thermo Scientific). Calibrated peak files were searched against the *S. epidermidis* RP62A proteins (Uniprot) by a SEQUEST search engine. Error tolerance on the fragment and precursor ions were +0.8 Da and +15 p.p.m, respectively. For peptide identification, a stringent cut-off value (0.5% false discovery rate, FDR) was used.

### Bioinformatics Tools

Gene ontology (GO) annotation and Kyoto Encyclopedia of Genes and Genomes (KEGG) pathway assignations were carried out to determine the function of differentially regulated genes, using STRING (version 10.5) with high confidence score of 0.7 (Carvalhais et al., 2014). Categories with a *p*-value ≤ 0.05 were set as statistically significant for gene enrichment. Differentially expressed hypothetical proteins were analyzed for putative functions using protein BLAST to search Pfam (protein families) domains in a Pfam database (version 31.0) and subcellular localization was predicted using PSORTb (Yu et al., 2010). The differentially expressed genes were also analyzed in the CELLO2GO^1^ to retrieve the sub-cellular localization and functional GO annotation against Gram-positive bacteria with an *E*-value 0.001 (Yu et al., 2014).

### Experimental Validation Using Quantitative Real-Time-PCR (qRT-PCR)

Twenty-one differentially expressed genes were selected for validation using qRT-PCR. The gene specific primers were designed using Primer3 software (version 0.4.0). The details of primers are listed in Supplementary Table 1. Total RNA was isolated as described above and cDNA was synthesized using Superscript III kit (Invitrogen Inc., United States) as per the manufacturer’s instructions. The qRT-PCR reaction was performed in 7500 Sequence Detection System (Applied Biosystems Inc., Foster, CA, United States). The mRNA expression patterns of selected genes were normalized against constitutively expressed two internal controls (16S*rRNA* and *rplU*) and quantified using 2^-ΔΔCt^ method. Three independent experiments were carried out in duplicate. Statistical significance was analyzed using unpaired Student *t*-test and considered significant at *P* < 0.05.

## Results

### Determination of α-MG Concentration for Transcriptomic and Proteomic Profiling Studies

The MIC and MBC of α-MG was found to be 1.25 and 5 μg/mL, respectively, which substantiates the results obtained from the previous study (Sivaranjani et al., 2017). Besides, Sivaranjani et al. (2017) have already reported the rapid bactericidal property of α-MG against *S. epidermidis* cells, where it displayed 6 log (4× MIC; 5 μg/mL) and 4 log (2× MIC; 2.5 μg/mL) reduction of viable count within 5 min of exposure time (Sivaranjani et al., 2017). Since transcriptional profiling is typically performed by the addition of antibacterial agents to mid-log phase cultures, spot assay was carried out to choose the concentration and duration of α-MG treatment. Based on the results, 0.7 MIC (0.875 μg/mL) of α-MG was chosen for 10 min and 30 min of antibacterial challenge with *S. epidermidis* (Supplementary Figure 1).

### Global Transcriptome and Proteome Response to α-MG Treatment

#### Transcriptome Analysis

To uncover the antibacterial mode of action of α-MG, RNA sequencing was performed from α-MG treated (0.7 MIC, 10 min) and untreated *S. epidermidis* RP62A cells. A total of 2,411 genes were identified under both the conditions, which correspond to 91.84% of *S. epidermidis* RP62A (2,625 genes in GenBank). Of 2,411 genes, the edgeR analysis revealed differential expression of 98 genes (4.06%) with ≥1.5-fold difference in log FC (log fold change) with *P* < 0.001, wherein 48 (1.9%) genes were upregulated while the remaining 50 (2.07%) genes were downregulated in response to α-MG treatment. The details of differentially regulated genes, their role and the log FC are listed in Table 1. Besides, 33.6% (33/98) of differentially regulated genes were encoding uncharacterized proteins. Hence, identities of protein families and sub-cellular localization of hypothetical proteins were predicted accordingly using Pfam and PSORTb (Supplementary Table 2). In addition, 30.3% (10/33), 27.2% (9/33), 3% (1/33), and 39.3% (13/33) of the hypothetical proteins were predicted to be localized in the cytoplasmic membrane-associated, cytoplasic, extracellular and location-unknown, respectively.

**Table 1:** List of differentially expressed genes of *S. epidermidis* treated with α-MG identified using RNA sequencing.

Gene ID	Gene	Product	log FC	*P*-value
SERP1987	*narG*	Respiratory nitrate reductase, alpha subunit	2.56	3.47 × 10^-23^
SERP0257	*adh*	Alcohol dehydrogenase, zinc-containing	1.95	2.12 × 10^-18^
SERP2080	*aldA*	Aldehyde dehydrogenase family protein	2.20	6.47 × 10^-18^
SERP2142	*ansP*	Amino acid permease family protein	2.70	3.98 × 10^-17^
SERP1784		Conserved hypothetical protein	2.12	5.32 × 10^-17^
SERP2327		Acetoin dehydrogenase, E3 component, dihydrolipoamide dehydrogenase	1.89	1.71 × 10^-14^
SERP2365	*pflA*	Pyruvate formate lyase activating enzyme	1.61	2.10 × 10^-13^
SERP0244		Oxidoreductase, aldo/ketoreductase family	2.04	3.91 × 10^-13^
SERP1986	*narH*	Respiratory nitrate reductase, beta subunit	1.74	4.21 × 10^-12^
SERP0245	*YdfJ*	Transporter, putative	233	9.35 × 10^-12^
SERP1783		Conserved hypothetical protein	1.66	1.21 × 10^-11^
SERP2112	*adh2*	Alcohol dehydrogenase, zinc-containing	1.79	3.85 × 10^-11^
SERP2412	*mqo-4*	Malate:quinone oxidoreductase	1.38	8.56 × 10^-11^
SERP2366	*pflB*	Formate acetyltransferase	1.40	1.44 × 10^-10^
SERP1990	*nirB*	Nitrite reductase [NAD(P)H], large subunit	2.17	1.56 × 10^-10^
SERP2324		Acetoin dehydrogenase, E2 component, dihydrolipoamide acetyltransferase	1.34	3.30 × 10^-10^
SEA0017	*yadH*	ABC transporter, permease protein	1.98	5.79 × 10^-10^
SERP2325	*acoB*	Acetoin dehydrogenase, E1 component, beta subunit	1.47	1.68 × 10^-09^
SEA0018	*ccmA*	ABC transporter, ATP-binding protein	2.02	3.86 × 10^-09^
SERP0419		Ribosomal subunit interface protein	1.23	8.43 × 10^-09^
SERP0897	*hom*	Homoserine dehydrogenase	3.25	1.20 × 10^-08^
SERP2029		Amino acid ABC transporter, amino acid-binding protein	1.50	1.80 × 10^-08^
SERP0270		Conserved hypothetical protein	1.27	2.15 × 10^-08^
SERP2326		Acetoin dehydrogenase, E1 component, alpha subunit	1.63	3.39 × 10^-08^
SERP0336		Conserved hypothetical protein	2.02	3.46 × 10^-08^
SERP1988	*sumT*	Uroporphyrinogen-III methylaseSirB, putative	1.85	1.02 × 10^-07^
SERP0216		Hexulose phosphate synthase, putative	1.31	1.05 × 10^-07^
SERP1782		Alkaline shock protein 23	1.15	1.11 × 10^-07^
SERP2469	*frmA*	Alcohol dehydrogenase, zinc-containing	2.19	2.78 × 10^-07^
SERP2031		Amino acid ABC transporter, ATP-binding protein	1.37	4.38 × 10^-07^
SERP1985	*narJ*	Respiratory nitrate reductase, delta subunit	2.07	9.87 × 10^-07^
SERP1980		Nitrite extrusion protein	2.11	2.91 × 10^-06^
SERP1476		Conserved hypothetical protein	3.30	3.84 × 10^-06^
SERP1484	*groEL*	Chaperonin, 60 kDa	0.95	4.08 × 10^-06^
SERP1483		Cell wall surface anchor family protein	0.99	4.23 × 10^-06^
SERP0665		Hypothetical protein	2.45	6.17 × 10^-06^
SERP1719	*glyA*	Serine hydroxymethyltransferase	1.00	6.68 × 10^-06^
SERP0348		Anion transporter family protein	1.39	7.93 × 10^-06^
SERP0903	*katA*	Catalase	0.93	8.30 × 10^-06^
SERP0444	*tpiA*	Triosephosphate isomerase	1.09	1.24 × 10^-05^
SERP1754		Conserved hypothetical protein	1.55	1.27 × 10^-05^
SERP1413		ThiJ/PfpI family protein	0.99	1.68 × 10^-05^
SERP1785		Alcohol dehydrogenase, zinc-containing	1.51	1.69 × 10^-05^
SERP1387	*fumC*	Fumarate hydratase, class II	0.94	2.42 × 10^-05^
SERP1790	*lacE*	PTS system, lactose-specific IIBC components	0.99	2.68 × 10^-05^
SERP0364	*saeS*	Sensor histidine kinase	0.90	3.83 × 10^-05^
SERP1789	*lacG*	6-phospho-beta-galactosidase	0.96	4.00 × 10^-05^
SERP0898	*thrC*	Threonine synthase	2.49	5.33 × 10^-05^
SERP1697	yidC2	Membrane protein insertase YidC 2	-1.70	1.60 × 10^-16^
SERP0679	ykyA	Conserved hypothetical protein	-1.32	1.27 × 10^-12^
SERP0518	dltA	D-alanine activating enzyme	-1.68	1.31 × 10^-11^
SERP1948	tcaA	tcaA protein	-1.40	1.74 × 10^-10^
SERP2241		Transcriptional regulator, putative	-3.43	1.19 × 10^-09^
SERP1088	accB	Acetyl-CoA carboxylase, biotin carboxyl carrier protein	-2.12	1.23 × 10^-09^
SERP1098	rhoD	Rhodanese like domain protein	-1.93	1.70 × 10^-09^
SERP0674	trkA	Potassium uptake protein TrkA	-2.01	1.94 × 10^-09^
SERP1738		Conserved hypothetical protein	-1.19	4.18 × 10^-09^
SERP0466		Cold shock protein, CSD family	-1.65	6.00 × 10^-09^
SERP1383	xre	Transcriptional regulator, Cro/CI family	-1.76	1.28 × 10^-08^
SERP2314	yceI	Conserved hypothetical protein	-1.26	1.36 × 10^-08^
SERP1761		Hypothetical protein	-1.79	2.01 × 10^-08^
SERP0541	yabR	General stress protein 13	-2.02	2.07 × 10^-08^
SERP0316	ykaA	Conserved hypothetical protein	-1.04	2.46 × 10^-08^
SERP2138		Immunodominant antigen A, putative	-1.05	3.22 × 10^-08^
SERP1472		Conserved hypothetical protein	-2.46	3.59 × 10^-08^
SERP0517		Conserved hypothetical protein	-2.32	7.67 × 10^-08^
SERP1381		Conserved hypothetical protein	-1.11	7.97 × 10^-08^
SERP2254		Conserved hypothetical protein	-1.61	1.89 × 10^-07^
SERP1330	*lytD*	*N*-acetylmuramoyl-L-alanine amidase, family 4	-1.11	2.01 × 10^-07^
SERP0057		Conserved hypothetical protein	-1.69	2.73 × 10^-07^
SERP0342	*norR*	Transcriptional regulator, MarR family	-1.06	3.59 × 10^-07^
SERP1896		Abortive infection family protein	-1.78	4.38 × 10^-07^
SERP0701		Conserved hypothetical protein	-1.20	4.43 × 10^-07^
SERP1772		Conserved hypothetical protein	-1.39	8.17 × 10^-07^
SERP0972		Cold shock protein, CSD family	-2.08	8.77 × 10^-07^
SERP0919	*mscL*	Large conductance mechanosensitive channel protein	-1.53	1.31 × 10^-06^
SERP0013		Conserved hypothetical protein	-1.33	1.54 × 10^-06^
SERP0001	*rpmH*	Ribosomal protein L34	-2.37	1.95 × 10^-06^
SERP2271		Conserved hypothetical protein	-2.07	2.95 × 10^-06^
SERP0557		Conserved hypothetical protein	-1.39	3.64 × 10^-06^
SERP1946		Transcriptional regulator, TetR family	-1.65	4.37 × 10^-06^
SERP1952		ABC transporter, permease protein	-1.28	4.39 × 10^-06^
SERP1154		Conserved hypothetical protein	-1.65	5.32 × 10^-06^
SERP1382		Conserved hypothetical protein	-2.25	6.90 × 10^-06^
SERP1694		Conserved hypothetical protein	-1.32	1.03 × 10^-05^
SERP1280	*nifS*	Aminotransferase, class V	-1.77	1.19 × 10^-05^
SERP0639		Conserved hypothetical protein	-1.53	1.74 × 10^-05^
SERP0717		Conserved hypothetical protein	-0.83	1.75 × 10^-05^
SERP1093	*efp*	Translation elongation factor P	-0.94	1.86 × 10^-05^
SERP2475		Conserved hypothetical protein	-1.40	2.38 × 10^-05^
SERP1215		Conserved hypothetical protein	-1.42	2.72 × 10^-05^
SERP1741	*luxS*	Autoinducer-2 production protein LuxS	-1.42	2.78 × 10^-05^
SERP2202		Conserved hypothetical protein	-0.85	3.11 × 10^-05^
SERP1664		Conserved hypothetical protein TIGR00150	-2.62	3.38 × 10^-05^
SERP1707		Conserved hypothetical protein	-2.62	3.38 × 10^-05^
SERP0048		Hypothetical protein	-2.16	4.31 × 10^-05^
SERP0297	*tagG*	tagG protein, teichoic acid ABC transporter protein, putative	-1.14	4.83 × 10^-05^
SERP1236	*hemA*	Glutamyl-tRNA reductase	-0.81	4.98 × 10^-05^


*Differentially expressed genes after α-MG treatment in *S. epidermidis* were identified using edgeR software with > 1.5 fold (given in log FC) and *P* < 0.001.*

**FIGURE 1:**
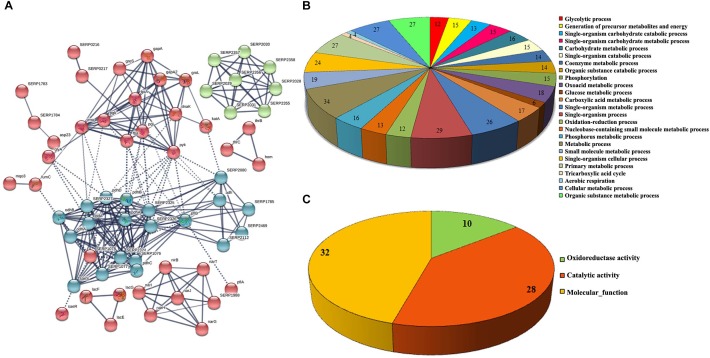
Interaction analysis of upregulated genes identified by RNA-sequencing. **(A)** Interactome map predicted using STRING_10.5_. The network nodes represent the proteins encoded by the genes. Edges represent protein-protein associations with high confidence score. The associations are meant to be specific and meaningful with associated proteins jointly contributing to a shared function. This does not necessarily mean that the proteins are physically binding each other, **(B)** significant biological process and **(C)** molecular function of upregulated genes.

**FIGURE 2:**
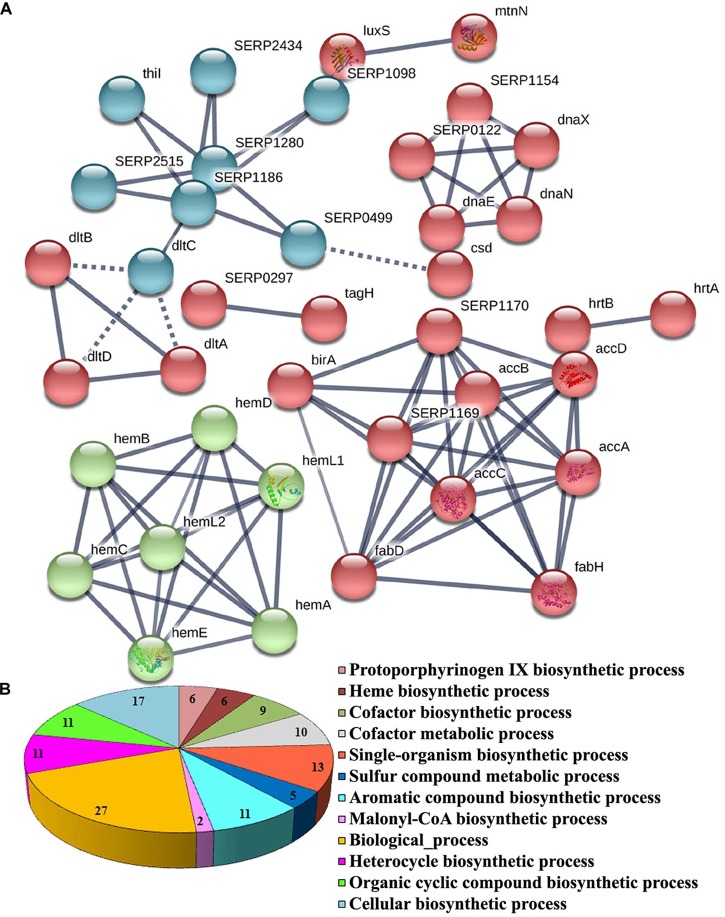
Significant GO annotation of downregulated genes identified by RNA-sequencing. **(A)** Interactome map predicted using STRING_10.5_. The network nodes represent the proteins encoded by the genes. Edges represent protein–protein associations with high confidence score. The associations are meant to be specific and meaningful with associated proteins jointly contributing to a shared function. This does not necessarily mean that the proteins are physically binding each other and **(B)** biological process of downregulated genes.

**FIGURE 3:**
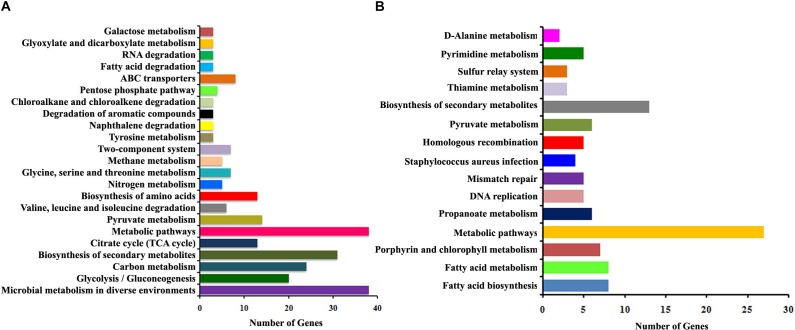
The histogram summarizing the enriched KEGG pathways of differentially expressed genes identified using RNA-sequencing. **(A)** Upregulated pathways and **(B)** downregulated pathways upon α-MG treatment.

The functional categorization for upregulated and downregulated genes was analyzed separately using STRING_10.5_. Subsequently, GO (biological process, molecular function and cellular component) analysis for differentially regulated genes were annotated manually and the interacting network maps are shown in Figure 1A, 2A. According to GO annotation, the upregulated genes were scattered among various biological processes which included oxidation-reduction, metabolic, phosphorylation, nucleobase-containing small molecule metabolic, tricarboxylic acid (TCA) cycle and aerobic respiration processes (Figure 1B). The predominant molecular functions of upregulated genes were catalytic activity, oxidoreductase activity and other molecular functions (Figure 1C). Further, in the KEGG pathway analysis, the upregulated genes were shown to be involved in glycolysis/gluconeogenesis, TCA cycle, carbon metabolism, nitrogen metabolism, pyruvate metabolism, aminoacid biosynthesis, ABC transporters, RNA and fatty-acid degradation pathways (Figure 3A). Meanwhile, GO annotation of downregulated genes were involved in various biological processes viz., protoporphyrinogen IX biosynthetic, heme-biosynthetic, cofactor biosynthetic, sulfur compound biosynthetic and malonyl-CoA biosynthetic processes (Figure 2B). In addition, the KEGG pathway analysis indicated that the downregulated genes are involved in several metabolic pathways that include biosynthesis of secondary metabolites, fatty-acid biosynthesis, DNA replication and mismatch repair (Figure 3B). Acetyl-CoA carboxylase complex (GO.0009317) was the only significant cellular component encoded by the downregulated genes. Furthermore, the CELLO2GO, used for functional GO annotation and sub-cellular localization for proteins encoded by differentially expressed genes (Supplementary Figure 2), substantiated the results obtained from STRING_10.5_ analysis. It has been reported to predict sub-cellular localization of proteins with 99.4% accuracy among Gram-positive bacteria (Yu et al., 2014). Among the proteins encoded by the 98 differentially expressed genes, 66.7, 18.9, 13.5, and 0.9% were predicted to be cytoplasmic, cytoplasmic membrane- associated, extracellular and cell wall-associated proteins, respectively.

#### Proteome Analysis

**FIGURE 4:**
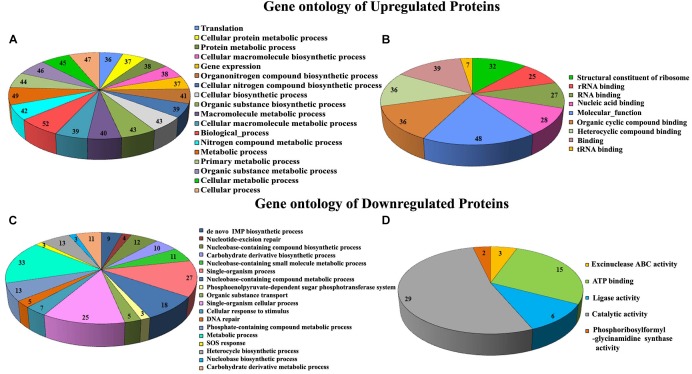
GO annotation of differentially expressed proteins of *S. epidermidis* upon α-MG treatment identified using LC-MS/MS analysis. Biological process **(A,C)** and molecular function **(B,D)**.

**FIGURE 5:**
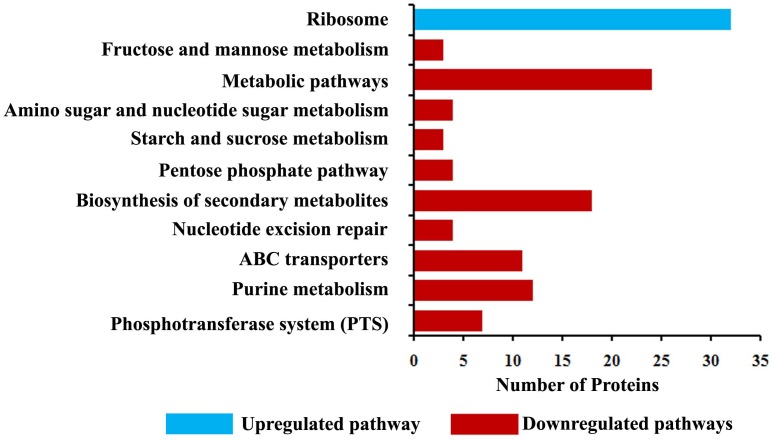
KEGG pathway analysis of differentially expressed proteins identified using LC-MS/MS analysis.

Proteomic profiling was employed to gain additional insights on the different cellular responses upon α-MG treatment. A total of 589 proteins were identified from all conditions, which corresponds to 23.51% of the predicted proteome (2505 proteins in UniProtKB database) of *S. epidermidis* RP62A. Prior to further analyses, Venn diagram (Supplementary Figure 3) was constructed to summarize the differences and similarities between proteins expressed in each conditions. It demonstrated that 492 proteins were expressed under all the conditions. Changes in proteome level were studied by comparing control cells with α-MG treated (0.7 MIC) cells at two different time points (10 and 30 min). Over all, differential expression (≥1.5-fold up- or down-regulation, *p* ≤ 0.05) of 89 proteins in response to α-MG treatment was observed: 34 proteins at 10 min (2 upregulated and 32 downregulated) and 70 proteins at 30 min (38 upregulated and 32 downregulated) (Table 2). However, only fifteen differentially expressed proteins (1 up- and 14 down-regulated) were found at both the time points. Of 89 proteins, 24 proteins were uncharacterized protein. The details of protein families and sub-cellular localization of uncharacterized proteins are listed in Supplementary Table 3. It was observed that most of the hypothetical proteins were localized in cytoplasmic membrane and cytoplasm (Supplementary Table 3). According to GO annotation, the upregulated and downregulated proteins are involved in various biological process and molecular function (Figure 4). The protein-protein interaction maps of upregulated and downregulated proteins are shown in Supplementary Figure 4. The KEGG pathway analysis predicted that the most strongly upregulated proteins are ribosomal proteins, and the most strongly downregulated proteins are involved in various metabolic pathways that include nucleotide excision repair, ABC transporters and phosphotransferase system (Figure 5). Besides, the GO annotation analysis of differentially regulated proteins using CELLO2GO is depicted in the Supplementary Figure 5. Among 89 differentially regulated proteins, 71, 15, and 11% were predicted to be cytoplasmic, membrane-associated, and extracellular proteins, respectively.

**Table 2:** List of differentially expressed proteins of *S. epidermidis* treated with α-MG identified using LC-MS/MS.

Gene ID	Gene	Product	0.7 MIC (10 min)		0.7 MIC (30 min)	
				
			FC	*P*-value	FC	*P*-value
SERP1356		Putative membrane protein insertion efficiency factor			9.87	0.04
SERP0651	*purC*	Phosphoribosylaminoimidazole-succinocarboxamide synthase			4.47	0.0003
SERP0045	*ssb*	Single-stranded DNA-binding protein			3.44	0.05
SERP0496	*sufC*	FeS assembly ATPase SufC			2.94	0.02
SERP0403		Transferrin receptor			2.78	0.04
SERP0300	*tagD*	Glycerol-3-phosphate cytidylyltransferase			2.59	0.05
SERP0946	*femA*	femA protein			2.33	0.06
SERP0039		Conserved hypothetical protein			2.23	0.002
SERP0679		Conserved hypothetical protein (putative cell wall binding lipoprotein)			2.23	0.04
SERP0170	*cysS*	Cysteinyl-tRNA synthetase			2.14	0.01
SERP1273		Universal stress protein family			2.14	0.02
SERP0826	*frr*	Ribosome recycling factor			2.12	0.01
SERP1781		Conserved hypothetical protein (IucA/IucC family)			2.06	0.05
SERP0690		Conserved hypothetical protein (protein of unknown function)			2.05	0.03
SERP2132	*copZ*	Heavy metal-binding protein, putative (Inorganic ion transport and metabolism)			1.98	0.003
SERP0435		Conserved hypothetical protein			1.98	0.003
SERP1431		Ferritin family protein (Inorganic ion transport and metabolism)			1.97	0.04
SERP1956	*RfaB*	Glycosyltransferase, group 1 family protein			1.96	0.04
SERP0139	*rplY*	Ribosomal protein L25			1.91	0.004
SERP0179	*rplA*	Ribosomal protein L1	1.66	0.04		
SERP0604		Conserved hypothetical protein			1.89	0.05
SERP1187		Bacterial luciferase family protein			1.88	0.03
SERP2046		Exopolyphosphatase			1.86	0.003
SERP1899	*murQ*	Glucokinase regulator-related protein			1.83	0.04
SERP0245	*YdfJ*	Transporter, putative (Uncharacterized membrane protein YdfJ)			1.83	0.04
SERP0587	*ppnK*	Conserved hypothetical protein			1.81	0.03
SERP1373		HIT family protein			1.81	0.03
SERP0791		Conserved hypothetical protein (alkaline shock protein (Asp23) family)			1.79	0.003
SERP2047	*ppk*	Polyphosphate kinase			1.75	0.02
SERP1132	*glyS*	Glycyl-tRNA synthetase			1.75	0.02
**SERP0527**		**NADH dehydrogenase, putative**	**1**.**53**	**0**.**05**	**1.77**	**0**.**01**
SERP0151	cysK	Chaperonin, 33 kDa			1.66	0.007
SERP1629		Hypothetical protein SERP1629			1.66	0.007
SERP0580	*pepF*	Oligoendopeptidase F			1.65	0.01
SERP0545	*rocD*	Ornithine aminotransferase			1.64	0.01
SERP0436	*clpP*	ATP-dependent Clp protease, proteolytic subunit ClpP			1.63	0.05
SERP0195	*ilvE*	Branched-chain amino acid aminotransferase			1.62	0.04
SERP0136	*spoVG*	spoVG protein			1.56	0.0005
SERP0188	*fusA*	Translation elongation factor G			1.53	0.007
SERP0217		SIS domain protein			-2.23	0.008
**SERP0427**	***uvrA***	**Excinuclease ABC, A subunit**	**INF**	**0**.**02**	**INF**	**0**.**02**
**SERP0312**	***ompR***	**DNA-binding response regulator**	**INF**	**0**.**003**	**INF**	**0**.**003**
SERP0525		Conserved hypothetical protein			INF	0.003
SERP0738		Phenol soluble modulin beta 1			INF	0.003
SERP0870		tRNA delta(2)-isopentenylpyrophosphatetransferase			INF	0.003
SERP0199		Hydrolase, haloaciddehalogenase-like family			INF	0.003
**SERP0764**	***pyrR***	**Pyrimidine operon regulatory protein**	**INF**	**0**.**003**	**INF**	**0**.**026**
**SERP0303**	***abcA***	**ABC transporter, ATP-binding/permease protein**	**INF**	**0**.**003**	**INF**	**0**.**008**
SERP0749		Cell division protein FtsQ, putative			INF	0.003
SERP2293	*icaA*	Intercellular adhesion protein A			INF	0.003
**SERP0409**		**Glycerate kinase family protein**	**INF**	**0**.**003**	**INF**	**0**.**003**
SERP1872	*ureE*	Urease accessory protein UreE			INF	0.003
SERP1900		PTS system, IIBC components			INF	0.003
**SERP1407**		**Conserved hypothetical protein**	**INF**	**0**.**003**	**INF**	**0**.**003**
**SERP1131**		**Conserved hypothetical protein**	**INF**	**0**.**003**	**INF**	**0**.**003**
**SERP0015**	***lolD***	**ABC transporter, ATP-binding protein**	**INF**	**0**.**003**	**INF**	**0**.**003**
**SERP2408**		**Conserved hypothetical protein**	**INF**	**0**.**003**	**INF**	**0**.**003**
**SERP2131**		**Cation-transporting ATPase,E1-E2 family**	**INF**	**0**.**003**	**INF**	**0**.**003**
**SERP2055**		**Phosphoglucomutase/phosphomannomutase family protein**	**INF**	**0**.**003**	**INF**	**0**.**003**
**SERP0323**		**Conserved hypothetical protein**	**INF**	**0**.**003**	**INF**	**0**.**003**
**SERP0241**		**Conserved hypothetical protein**	**INF**	**0**.**003**	**INF**	**0**.**003**
SERP1628		Conserved hypothetical protein			INF	0.003
SERP0419	*raiA*	Ribosomal subunit interface protein	5.8	0.04		
SERP0742		Conserved hypothetical protein	INF	0.03		
SERP0846		Peptidase, M16 family	INF	0.02		
SERP0246		ABC transporter, substrate-binding protein, putative	INF	0.008		
SERP0447		Conserved hypothetical protein	INF	0.003		
SERP0512		Conserved hypothetical protein	INF	0.003		
**SERP0652**	***purS***	**Phosphoribosylformylglycinamidine synthase, PurS protein**	**INF**	**0**.**003**	**INF**	**0**.**003**
SERP1704		ywpF protein	INF	0.003		
SERP0206		Azoreductase	INF	0.008		
SERP0423		HD domain protein, putative	INF	0.003		
SERP0654	*purL*	Phosphoribosylformylglycinamidine synthase II	INF	0.03		
SERP0866	*glpF*	Glycerol uptake facilitator protein	INF	0.05		
SERP2378	*ccmA*	ABC transporter, ATP-binding protein	INF	0.003		
SERP0774		Conserved hypothetical protein	INF	0.003		
SERP0297		tagG protein, teichoic acid ABC transporter protein, putative	INF	0.003		
SERP0891		Thermonuclease precursor family protein	INF	0.003		
SERP1839	*gdh*	Glucose 1-dehydrogenase	INF	0.05		
SERP0433		Conserved hypothetical protein	INF	0.003		
SERP0099		ABC transporter, substrate-binding protein	INF	0.003		
SERP0141	*mfd*	Transcription-repair coupling factor			INF	0.001
SERP0831	*polC*	DNA polymerase III, alpha subunit, Gram-positive type			INF	0.003
SERP0240		Phosphomevalonate kinase			INF	0.003
SERP0358	*fruK*	1-phosphofructokinase			INF	0.003
SERP0385		ABC transporter, ATP-binding protein			INF	0.003
SERP1324	*putA*	Proline dehydrogenase			INF	0.058
SERP0252		Conserved hypothetical protein			INF	0.058
SERP1901		Phosphosugar-binding transcriptional regulator, RpiR family			INF	0.003


*INF, these proteins could not be calculated because the proteins in query was below the detection threshold level for one of the conditions. The proteins expressed in both conditions are marked in bold type.*

### Validation of RNA-Sequencing and LC-MS/MS Data by qRT-PCR

**FIGURE 6:**
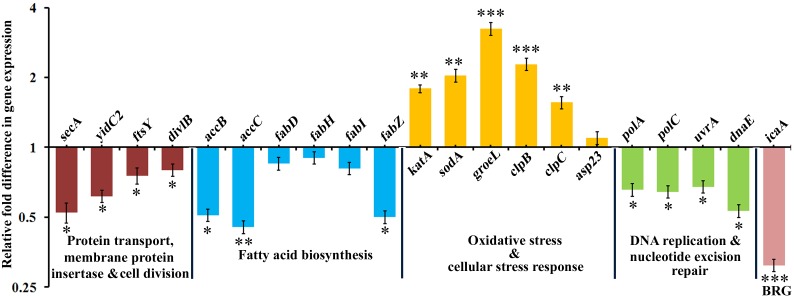
The qRT-PCR validation of differentially expressed genes which are randomly selected based on transcriptomic and proteomic approaches. BRG stands for biofilm regulatory gene. The data represented the mean ± standard deviation of three independent experiments. The statistical significance of data was determined by unpaired Student’s *t*-test. ^∗^*P <* 0.05, ^∗∗^*P <* 0.005, ^∗∗∗^*P <* 0.001.

The transcriptomic and proteomic results were further validated using qRT-PCR. qRT-PCR was performed with RNA isolated from bacteria under the similar experimental conditions used for the RNA-sequencing and LC-MS/MS analysis experiments. A total of 21 genes were selected randomly based on the functional categorization and KEGG pathway analysis. The obtained results were consistent with the transcriptomic and proteomic data (Figure 6), which not only validated the expression profiles of the selected genes, but also substantiated the reliability and accuracy of our transcriptome and proteome analyses. Based on the integrated transcriptome and proteome analyses and further validation through qRT-PCR, it was speculated that α-MG targets *S. epidermidis* through multifarious pathways viz., by principally targeting the cytoplasmic membrane integrity and by subsequently affecting fatty acid biosynthesis, DNA replication, DNA repair machinery, oxidative and cellular stress responses.

## Discussion

The combination of transcriptome and proteome analyses demonstrated a panoramic view of the antibacterial mechanism of α-MG on *S. epidermidis*. Koh et al. (2013) reported that the rapid bactericidal action of α-MG is comparable with cell membrane lytic cationic antimicrobial peptides (CAMPs). The presence of carbonyl and hydroxyl groups might confer the negative charge to α-MG (Ahmad et al., 2013). Unlike conventional antibiotics that interact with specific targets, many CAMPs bind to anionic bacterial surface and perturb the cyotplasmic membrane (Hancock, 2001). In addition, α-MG is known to interact with transmembrane precursor proteins via hydrogen bonding (Wang et al., 2012). In parallel with this report, α-MG strongly downregulated the *yidC2* gene (Table 1), which is involved in the integration of nascent membrane protein with dependent from or independent from the Sec-translocase (van der Laan et al., 2003). The proper membrane biogenesis depends on YidC, wherein it acts as a protein insertase, facilitates protein folding, ensues proper topology and an assembly factor for transmembrane proteins (Wang and Dalbey, 2011). The downregulated gene *yidC2* (encoding a membrane protein insertase) was found to interact with the Sec pathway proteins such as SecA, SecY and FtsY, and the cell division protein FtsQ (encoded by *divlB*). The data from qRT-PCR revealed that *secA, ftsY* and *divlB* were downregulated upon α-MG treatment (Figure 6). Though data on the Sec system of *Staphylococcus* spp. is scarce, *secA* has been shown to be essential for cell viability (Ji et al., 2001; Jin et al., 2015). The small analogs of Rose Bengal inhibited the *secA1* and *secA2* of *S. aureus* thereby reducing the toxin secretion and bypassing the negative effect of efflux pumps (Ji et al., 2001). In addition, MscL (large conductance mechanosensitive channel protein) plays a vital role in protecting cells from osmotic downshock (Booth and Louis, 1999), which was downregulated upon α-MG treatment (Table 1). Facey et al. (2007) suggested that signal recognition particle (SRP) plays an important role in targeting of MscL protein to the cytoplasmic membrane and hence, the deletion of SRP receptor FtsY had a significant effect on the insertion of MscL. Moreover, the depletion of YidC2 inhibits the translocation of protein across the membrane. This confirms that downregulation of YidC2 and FtsY expression could inhibit the insertion of membrane protein MscL. Hence, the downregulation of these very important cytoplasmic membrane associated genes/proteins further corroborate that α-MG elicits bacterial killing by affecting the integrity of cytoplasmic membrane.

α-MG induced the expression level of certain heat-shock proteins (ClpB, ClpC, and GroEL) that ultimately rescue the cells from the lethal effect of α-MG (Table 1 and Figure 6). In parallel with our observation, the enhanced expression of the *groES* and *groEL* genes was observed upon oxacillin treatment, suggesting that upregulation of these heat-shock protein encoding genes is an integral part of cell wall active-antibiotic stress response (Singh et al., 2001). Under stress conditions, the chaperons and heat-shock proteins are capable of reactivating and refolding denatured proteins. Acyldepsipeptides, a new class of antibiotics, activate ClpP to degrade the cell division protein FtsZ, thereby arresting cell division (Sass et al., 2011). In line with this report, it was hypothesized that α-MG decrease the expression level of cell division related proteins (FtsY and DivlB) by increasing the Clp protease activity. Further, α-MG treatment increased the expression level of *asp23* encoding the alkaline shock protein (Table 1 and Figure 6). In *S. aureus*, the transcription of *asp23* is used as a marker for the activity of alternative sigma factor Sigma^B^, which responds to various stress conditions by rapidly increasing its activity (Bischoff et al., 2001; Senn et al., 2005; Cebrian et al., 2009). Further, increased expression levels of the oxidative stress response genes, *katA* and *sodA* (encoding catalase and superoxide dismutase, respectively) were observed on α-MG treatment (Table 1 and Figure 6).

In addition to cytoplasmic membrane perturbation, α-MG seems to target very basic metabolic processes in order to kill the bacteria. Enzymes responsible for DNA replication play an indispensable role in cell growth and are therefore appropriate targets for antimicrobial agents. DNA polymerase I is encoded by the gene *polA* and two other important DNA replication specific enzymes, DNA polymerase III C and DNA polymerase III E are encoded by *polC* and *dnaE*. Both *polC* and *dnaE* are essential for chromosomal DNA replication in Gram-positive bacteria (Dervyn et al., 2001; Inoue et al., 2001). As reported by Inoue et al. (2001), inactivation of either *polC* or *dnaE* has a bactericidal effect on *S. aureus*. In addition, the downregulated genes *uvrA* and *mfd* have been shown to be involved in nucleotide excision repair machinery. UvrA detects the damaged DNA and the transcription repair coupling factor mfd attract the DNA-excision repair machinery to the damaged DNA (Ambur et al., 2009). Our transcriptomic and proteomic data shows the downregulation of *polA, polC, dnaE, uvrA*, and *mfd* genes upon α-MG treatment (Table 2 and Figure 6). Besides, the transcriptomic data shows the downregulation of the *accB* gene encoding a protein that interacts with AccC, AccB, AccA, AccD, FabF, FabG, FabH, FabI, and FabZ proteins (Table 1). These proteins are involved in fatty acid biosynthetic pathway (FASII), in which AccB is involved in the first committed step of FASII, catalyzing the formation of malonyl-CoA from acetyl-CoA. The qRT-PCR analysis also demonstrated the downregulation of all the selected genes involved in FASII pathway (Figure 6). In line with our report, platensimycin and platencin were shown to inhibit key FASII enzymes such as FabB/F and/or FabH against *S. aureus, Streptococcus pneumonia, Enterococcus faecalis* and *Enterococcus faecium*, which resulted in bacterial growth inhibition (Wang et al., 2006, 2007; Wright and Reynolds, 2007). In general, it is hypothesized that α-MG subsided the DNA replication, DNA repair machinery and fatty acid biosynthesis pathway which might further augment the bactericidal property of α-MG against *S. epidermidis.*

Furthermore, our transcriptomic and proteomic data shows the downregulation of *tagG* (encoding the teichoic acid ABC transporter protein) known to be involved in teichoic acids biosynthesis pathway (Tables 1, 2). The cell wall of gram-positive bacteria is mainly composed of peptidoglycan and Teichoic acids, both of which are important for maintaining the shape and structural integrity of the bacterial cell. Teichoic acids are anomeric polyglycerophosphate chains, which are exported via TagGH, a two component ABC transporter and then, either coupled with peptidoglycan (wall teichoic acids) or anchored from the cytoplasmic membrane (lipoteichoic acids). However, the D-alanylation modification regulates the interactions between bacterial cell membrane and CAMPs. For instance, increased D-alanylation of TAs provides resistance to CAMPs, possibly by increasing positive surface charge density. Our transcriptomic data suggested that the expression of *dltA* was clearly downregulated upon α-MG treatment (Table 1). The D-alanylation of TAs is directed by the *dlt* operon, where DltA begins the biosynthetic pathway by activating the D-alanine from aminoacyl adenylate. May et al. (2005) reported the analog of D-Ala aminoacyl adenylate as the inhibitor of DltA, that enhanced the susceptibility of *Bacillus subtilis* against cationic antibiotic vancomycin. In line with this report, our previous study signifies that the continuous exposure of α-MG does not induce resistance in planktonic as well as biofilm cells of *S. epidermidis*. This is possibly due to the downregulation of *dltA* which in turn averts positive surface charge density on bacterial membrane and facilitates the interaction between α-MG and bacterial cell membrane. Moreover, *dltA* mutant strain of *S. aureus* is unable to form biofilm on glass or polystyrene, which has been attributed by the inhibition of initial adherence and not accumulation, as the levels of polysaccharide intercellular adhesin (PIA) were unaltered (Gross et al., 2001). *S. epidermidis* and *S. aureus* have shown to possess *icaABCD* operon, wherein *icaA* encodes for *N*-acetylglucosaminyltransferase, an enzyme which is important for synthesis of PIA. The proteomic and qRT-PCR analyses showed downregulation of *icaA* which goes in parallel with our previous report (Sivaranjani et al., 2017), in which a less pronounced biofilm inhibition was observed at sub-MIC of α-MG. Of note, the mode of action of α-MG appears to involve compromising the cytoplasmic membrane integrity of Gram-positive bacteria. The cytoplasmic membrane perturbing ability of α-MG may also add credits in restricting the acquisition of bacterial resistance, as there is evidence suggesting that bacteria do not easily acquire resistance against membrane active antibacterial agents (Przybylski et al., 1998; Ooi et al., 2009).

## Conclusion

**FIGURE 7:**
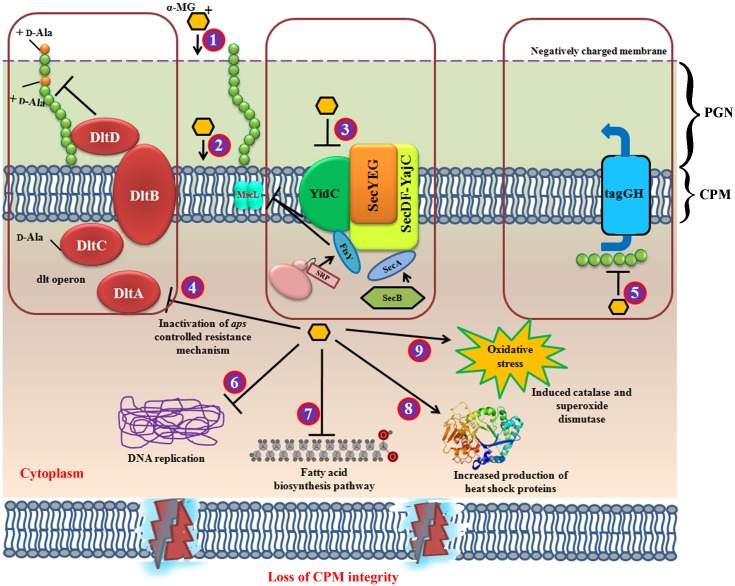
Schematic representation of multifarious antibacterial mode of action for α-MG. **(1)** α-MG shows electrostatic interaction with negatively charged bacterial membrane, where it binds with the bacterial inner membrane **(2)** in which, the strong hydrophobic interaction with lipid alkyl chain of cytoplasmic membrane might be the major driving force for the rapid bactericidal property. Further, α-MG interacts with the transmembrane precursor proteins via hydrogen bonding. **(3)** The downregulation of YidC2, SecA, FtsY, and MscL has evidenced that α-MG thwart the cytoplasmic membrane integrity. **(4)** The downregulation of dltA inhibits D-alanylation of anomeric teichoic acids and hence, the development of resistance against α-MG has been aborted. **(5)** TagG, a part of two component ABC transporter which exports the anomeric polyglycerophosphate chains (TAs) from cytoplasm. Inhibition of tagG shows that teichoic acid biosynthetic pathway has also been affected, because tagG is localized in cytoplasmic membrane. This might possibly due to the loss of cytoplasmic membrane integrity which resulted in decreased export of teichoic acids from cytoplasm. **(6)** DNA replication and mismatch repair mechanism has been downregulated upon α-MG treatment. **(7)** α-MG also inhibited fatty acid biosynthesis pathway **(8)** α-MG also enhanced the expression of heat shock proteins. **(9)** α-MG has also shown to increase the genes involved in oxidative stress response. CPM, cytoplasmic membrane; PGN, peptidoglycan.

Based on the integrated high throughput transcriptomic and proteomic analyses, the antibacterial mode of action of α-MG was identified to be more complex that targets multiple metabolic pathways. It was speculated that the principal target of α-MG seems to be cytoplasmic membrane which further orchestrate the downregulation of genes important for various metabolic pathways. α-MG downregulated the genes involved in FASII pathway, cell division, DNA replication, homologous recombination, mismatch repair, resistance development, biofilm, oxidative stress and cellular stress response, which are believed to be secondary targets of α-MG (Figure 7). As α-MG targets several metabolic pathways, resistance development by the bacterium will be minimal and thus α-MG may be further exploited for clinical trials. To our knowledge, this is one of the very first reports that potentiate the antibacterial activity of α-MG through an integrated transcriptomic and proteomic approach.

In addition, as α-MG targets multifarious pathway, further in depth analysis viz., mutational and *in vivo* studies on each pathway can be evaluated to identify the primary and secondary targets of α-MG. This will pave the way to develop α-MG as one of the vital drug candidates to treat the infections caused by *S. epidermidis*. Further, combination of any known bioactive compounds or with effective antibiotics not only increases the activity of known bioactive compounds but also can possibly assist the clinical development of these bioactive compounds. Further, combinatorial therapy avoids the resistant development by the bacterial pathogens as it involves the usage of lower/shorter dosing regimens. Hence, combination of α-MG with other well-known phytochemicals or conventional antibiotics can be carried out to evaluate the bactericidal property on mature biofilms.

## Author Contributions

MgS and CA designed the study. MgS, KL, and MkS performed the experiments and interpreted the data. MkS, PS, AR, and SP contributed to materials and reagents. MgS and CA drafted the manuscript. All authors approved the final manuscript.

## Conflict of Interest Statement

The authors declare that the research was conducted in the absence of any commercial or financial relationships that could be construed as a potential conflict of interest.

## Footnotes

1 http://cello.life.nctu.edu.tw/cello2go/
