# Supplementary material for: Deciphering the Antibacterial Mode of Action of Alpha-Mangostin on Staphylococcus epidermidis RP62A Through an Integrated Transcriptomic and Proteomic Approach
Source: Front Microbiol. 2019 Feb 6;10:150. doi: 10.3389/fmicb.2019.00150 (PMC6372523; doi:10.3389/fmicb.2019.00150)
Supplement: Supplementary file 1 [file Data_Sheet_1.doc]

**Supplementary FIG. 1.** Spot assay of *S. epidermidis* RP62A in the absence (control) and presence (1.25 µg/mL to 0.375 µg/mL) of α-MG. MIC (1.25 µg/mL) of α-MG shown approximately ˂ 2 log reduction in *S. epidermidis* growth within 10 min. Hence, 0.7 MIC (0.875 µg/mL) of α-MG was used for antibacterial challenge with *S. epidermidis*.


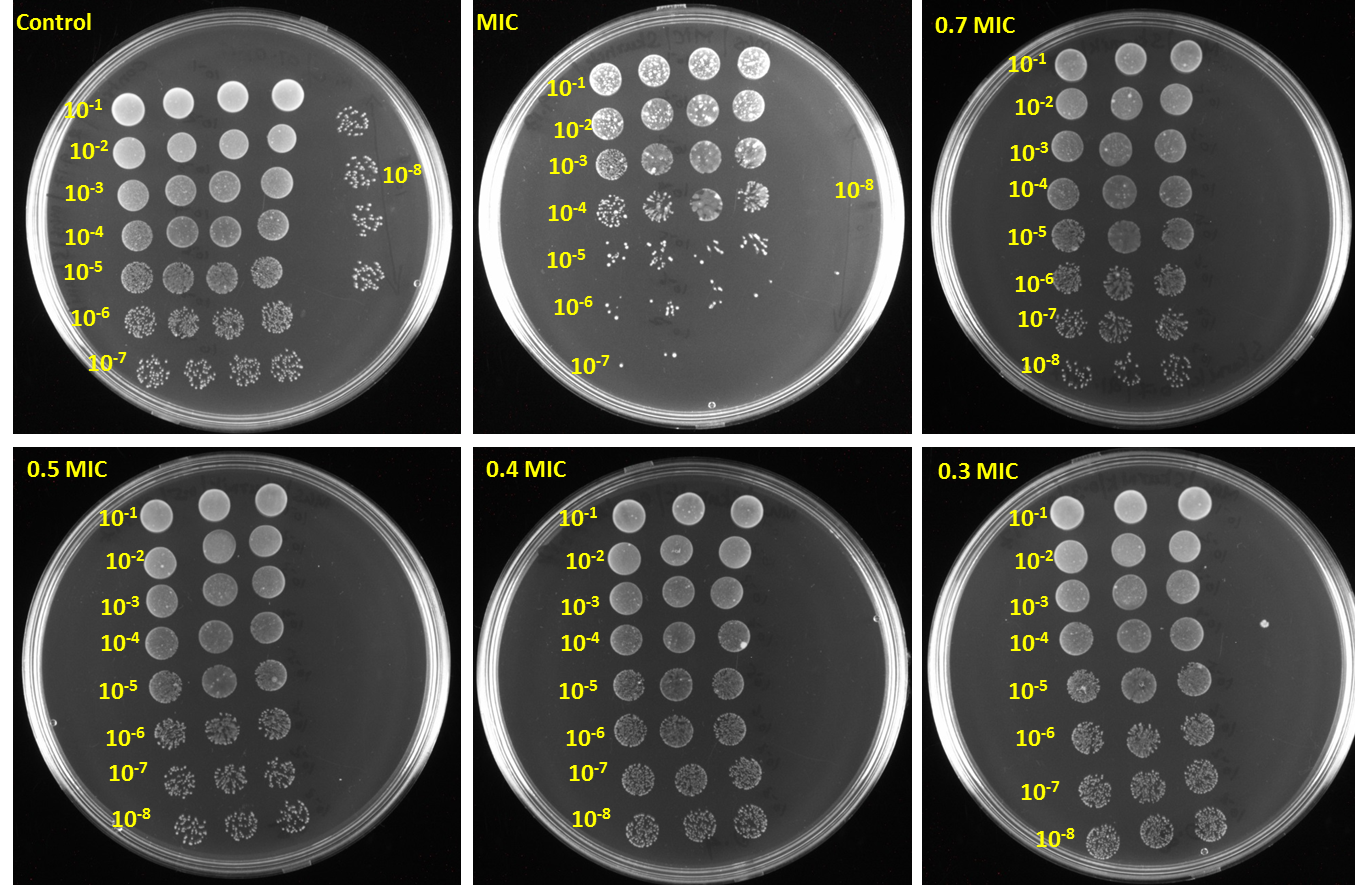


**Supplementary FIG. 2.** CELLO2GO analysis. GO annotation of differentially expressed genes upon α-MG treatment which are identified using RNA-sequencing. Biological process (A), Molecular function (B) and Cellular component (C).


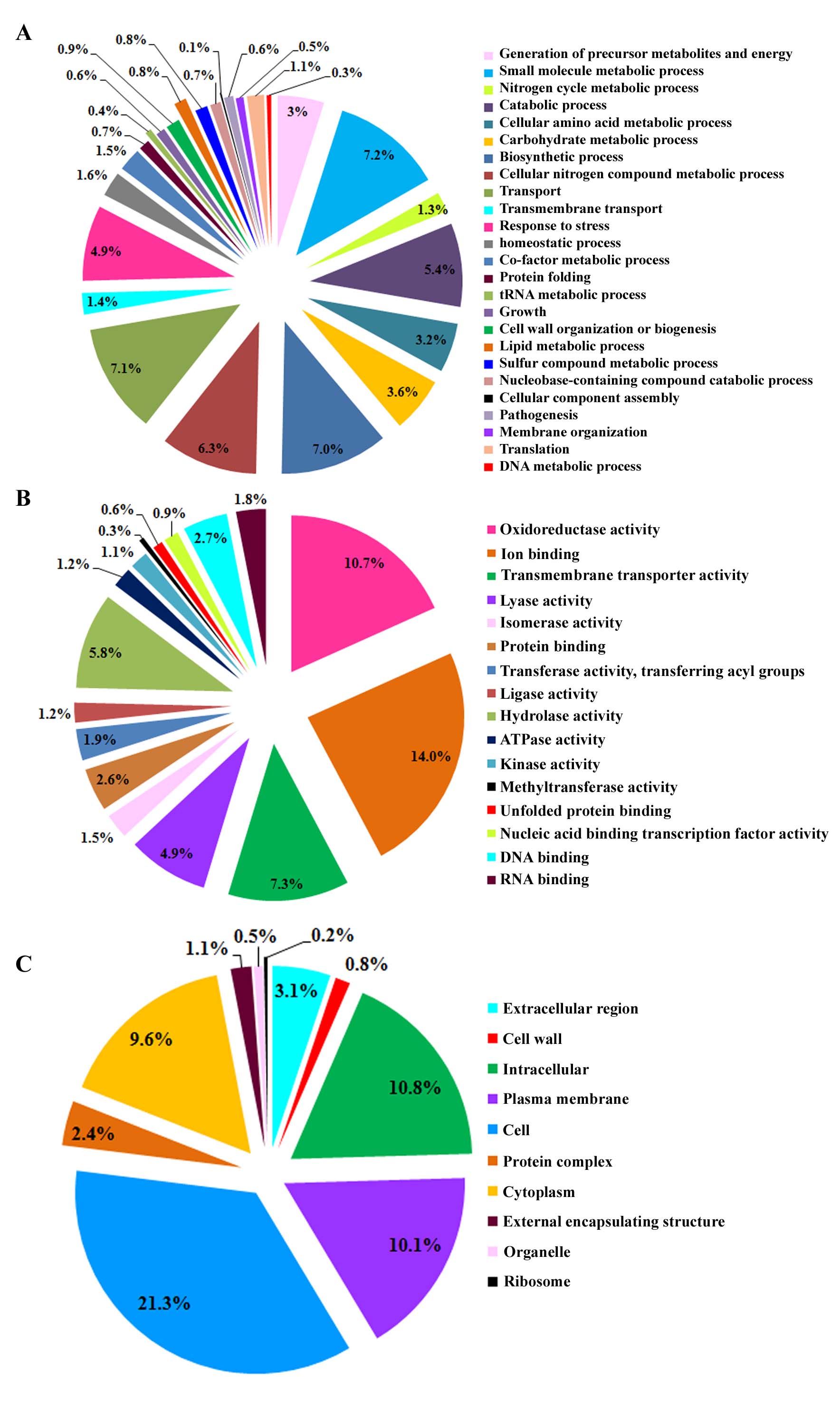


**Supplementary FIG. 3.** Venn diagram representing the overlapped proteins identified by LC-MS/MS in *S. epidermidis* RP62A with presence and absence α-MG (0.7 MIC for 10 min and 30 min of exposure).


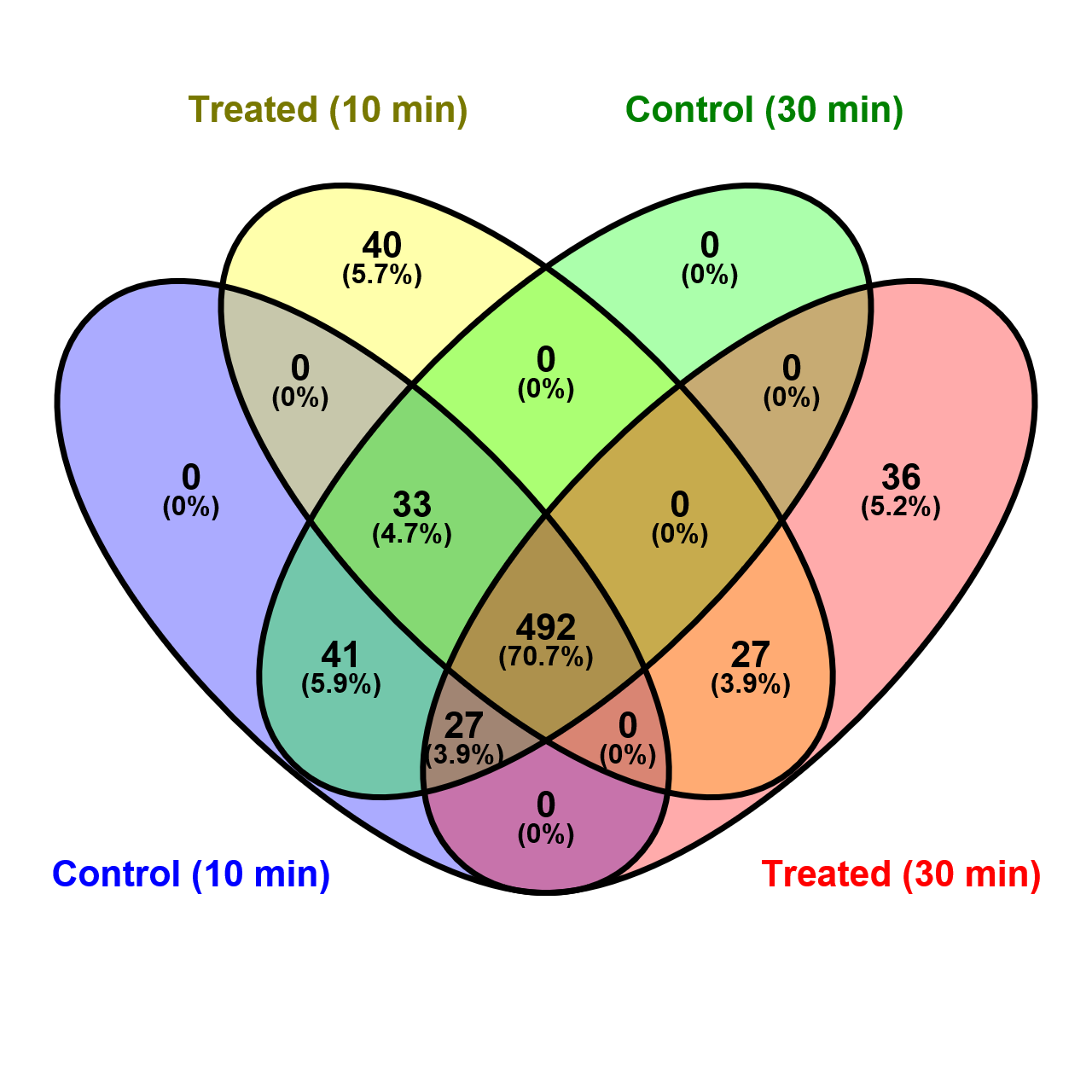


**Supplementary FIG. 4.** STRING_10.5_ predicted the protein-protein interactions (high confidence score 0.700) of upregulated (A) and downregulated (B) proteins of *S. epidermidis* RP62A upon α-MG treatment.

**
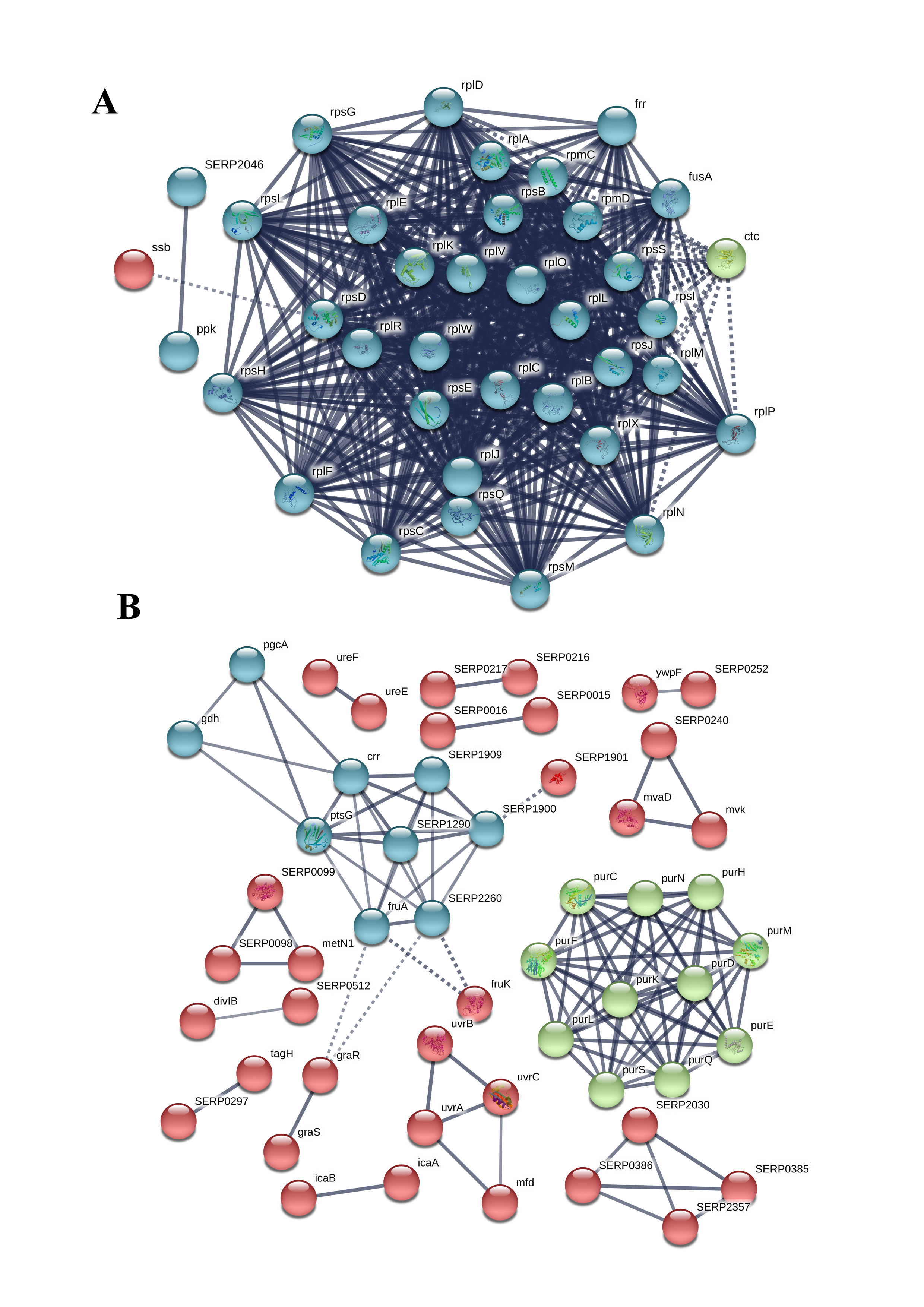
**

**Supplementary FIG. 5.** CELLO2GO analysis. GO annotation of differentially expressed proteins upon α-MG treatment that were identified using LC-MS/MS. Biological process (A), Molecular function (B) and Cellular component (C).

**
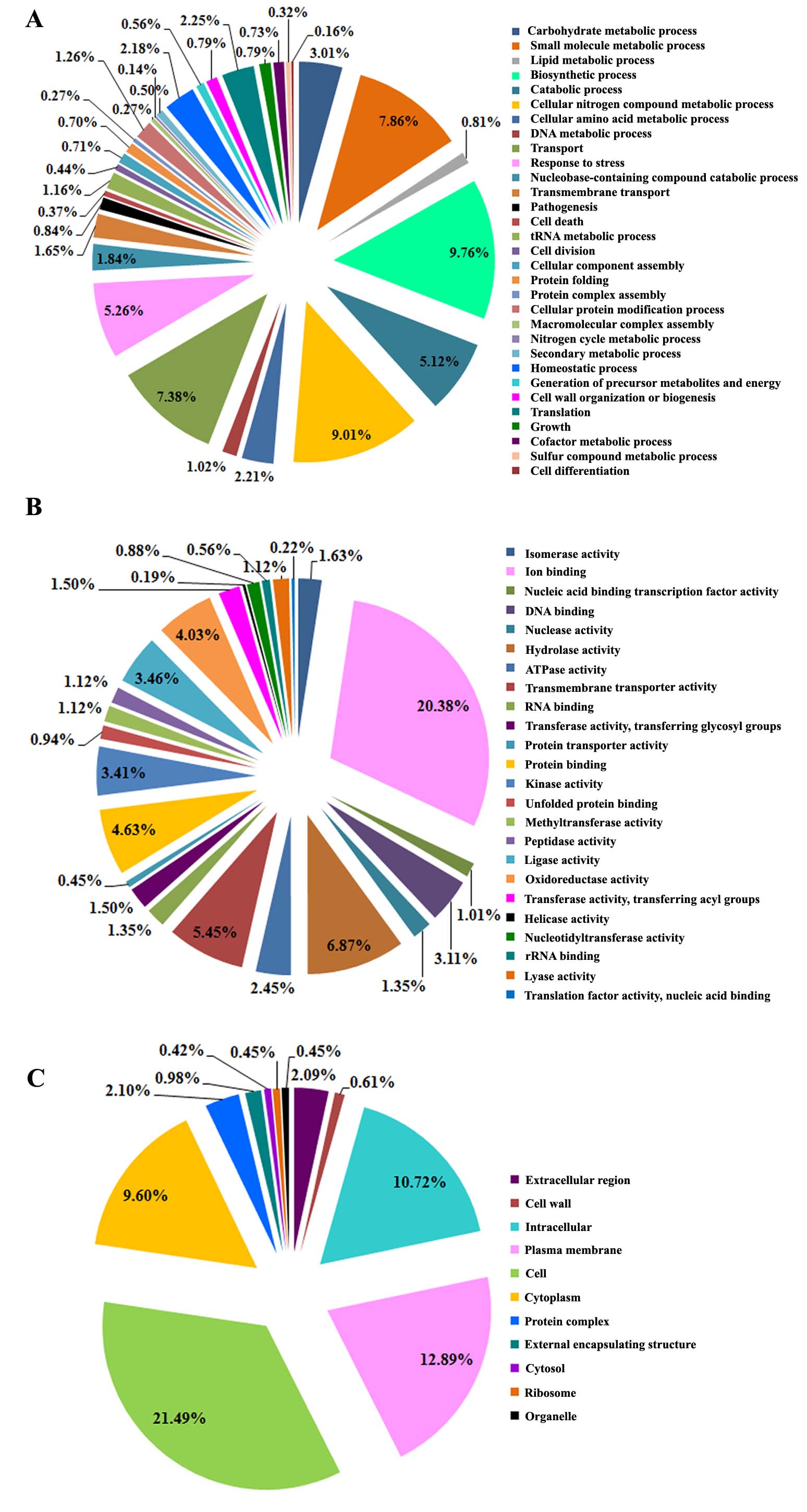
**
